# Apple Intrinsic Factors Modulating the Global Regulator, LaeA, the Patulin Gene Cluster and Patulin Accumulation During Fruit Colonization by *Penicillium expansum*

**DOI:** 10.3389/fpls.2018.01094

**Published:** 2018-07-27

**Authors:** Dilip Kumar, Joanna Tannous, Edward Sionov, Nancy Keller, Dov Prusky

**Affiliations:** ^1^Department of Postharvest Science of Fresh Produce, Agricultural Research Organization, The Volcani Center, Bet Dagan, Israel; ^2^Department of Medical Microbiology and Immunology, University of Wisconsin—Madison, Madison, WI, United States; ^3^Department of Bacteriology, University of Wisconsin—Madison, Madison, WI, United States; ^4^Department of Food Storage, Agricultural Research Organization, The Volcani Center, Bet Dagan, Israel; ^5^College of Food Science and Engineering, Gansu Agricultural University, Lanzhou, China

**Keywords:** patulin, LaeA activation in fruits, transcript activation by apple factors, mycotoxin accumulation in fruits, regulation of mycotoxin synthesis

## Abstract

The mycotoxin patulin is produced in colonized tissue by *Penicillium expansum* during storage of apples and is significantly affected by environmental factors that contribute to its accumulation. Few reports have, however, examined the effect of natural intrinsic factors associated with the fruit on the production of patulin. Here, we find that with advancing maturity, Golden Delicious apples show increased concentrations of total soluble solids (TSS) from 14 to 17% associated with the increased expression of the global transcription factor involved in regulation of secondary metabolite biosynthesis in filamentous fungi, *laeA* expression and patulin accumulation. However, the apple cultivar Granny Smith, with similar TSS values but differing in pH levels and malic acid concentrations, showed reduced expression levels of *laeA* and the patulin biosynthesis gene cluster (*pat* genes) and patulin accumulation, suggesting a complexity of host factors contribution to patulin accumulation during *P. expansum* colonization. To start elucidating these apple intrinsic factors, we examined their *in vitro* impact on *laeA* and *pat* gene expression concomitant with patulin synthesis. Increasing sucrose concentrations from 15 to 175 mM repressed *laeA* and *pat* gene expression and patulin production. However, this affect was modified and often reversed and sometimes accentuated by changes in pH, or the addition of malic acid or the major apple phenolic compounds, chlorogenic acid and epicatechin. While the increase in malic acid from 0 to 1% increased *laeA* and *pat* gene expression, the decrease in pH from 3.5 to 2.5 reduced their expression. Also the increased *laeA* and *pat* genes expressions at increasing epicatechin concentrations from 0 to 1 mM, was reversed by increasing sucrose concentrations, all together suggesting the complexity of the interactions *in vivo*.

## Introduction

*Penicillium expansum* is a destructive phytopathogen, capable of causing decay in many deciduous fruits during post-harvest handling and storage (Prusky et al., 2004). The fungus produces large amounts of the secondary metabolite, patulin, a relatively uncomplex lactone. Patulin is produced by several species belonging to the genera *Penicillium*, *Aspergillus*, *Paecilomyces*, and *Byssochlamys* (Puel et al., 2007). *P. expansum* is generally regarded as the major producer of patulin (McKinley and Carlton, 1991; Andersen et al., 2004; Pitt et al., 2009). Apple juice and other derived fruit products from *P. expansum* infected apples are the major source of patulin. Long-term exposure to patulin contaminated fruit juices can cause serious health issues as patulin is believed to be mutagenic, neurotoxic, genotoxic, and immunotoxic to animals (Wouters and Speijers, 1996; Moake et al., 2005).

Given the importance of preventing toxin accumulation in fruit, several environmental conditions that contribute to patulin accumulation during fruit colonization have been analyzed. These include temperature, ambient pH, and water activity (Schmidt-Heydt et al., 2008). Patulin production is also dependent on a variety of complex interactions including the geographical location where fruits are grown and harvested, the pathogen load on the fruit, the fungal strain, the fruit type and cultivar, ripening, and other physiological properties of fruits (Spotts et al., 1999; de Souza Sant’Ana et al., 2008; Morales et al., 2008; Jan et al., 2012). Moreover, storage conditions were found to have a great effect on patulin accumulation. This was perceived when fruits were stored at ultralow oxygen levels, conditions that delayed fruit ripening and therefore reduced patulin accumulation (Morales et al., 2008). However, in a study by Baert et al. (2007) whereas controlled atmospheres did not affect growth of *P. expansum*, they did reduce patulin production, suggesting that environmental conditions may affect patulin biosynthesis.

Carbon sources also affect patulin production. Davis and Diener (1968) and Zong et al. (2015) analyzed conditions *in vitro* finding that different carbon sources in defined media could strongly influence patulin production in *P. expansum*. Maltose, glucose, fructose, mannose, sucrose, and starch were favorable C sources for patulin biosynthesis. Other carbon sources, such as citrus pectin, lactose, malic acid, and cellulose showed very low levels of patulin accumulation when added in defined media. The same study examined the repression of patulin induction by ammonium sulfate and ammonium chloride at a concentration of 30 mM, in *Penicillium urticae* (syn. *Penicillium griseofulvum*) (Rollins and Gaucher, 1994). However, in a different study, the same ammonium chloride, but in a reduced concentration (22 μM only) actually enhanced patulin accumulation by *P. expansum* (Barad et al., 2015).

The pH levels were also found to modulate patulin accumulation during fruit colonization. While patulin accumulation by *P. expansum* was found to be related to acidification conditions (Prusky et al., 2004; Li et al., 2009, 2012, 2015; Barad et al., 2014), it was Zong et al. (2015) that concluded that acidic conditions (pH 3, 4, or 5) are more favorable for patulin production than alkaline conditions, similarly to other reports on aflatoxin (Shih and Marth, 1974; Keller et al., 1997) and trichothecenes (Merhej et al., 2011).

More recently with advances in *P. expansum* genetics, studies have identified several genes required for patulin synthesis including genes in the patulin gene cluster, *patE*, *patG*, *patK*, *patL*, *patH*, and *patI* (Beck et al., 1990; Wang et al., 1991; Fedeshko, 1992; Dombrink-Kurtzman and Engberg, 2006; Dombrink-Kurtzman, 2007; Artigot et al., 2009; Snini et al., 2014, 2016; Li et al., 2015; Tannous et al., 2017), the global regulator of secondary metabolism, LaeA (Kumar et al., 2017), the transcription factor PeSte12 (Sánchez-Torres et al., 2018), the SdnB subunit of respiratory complex II (Malandrakis et al., 2017) and LysM effectors (Levin et al., 2017).

While a significant amount of work was carried out to understand factors that modulate patulin accumulation *in vitro* as above (Tannous et al., 2015), the host intrinsic factors that modulate patulin accumulation are barely studied (McCallum et al., 2002; Marín et al., 2006). Here, we examine a series of intrinsic critical host factors that change during fruit ripening—including sugars, organic acids, pH, and phenols—and present their impact on patulin synthesis and *laeA* expression. We suggest in the present work that intrinsic apple factors differentially contribute to the complex activation of the patulin gene cluster and contribute to the accumulation of patulin in colonized fruits.

## Results

### Fruit Maturation Affects Differential Expression of *pat* Genes and Consequent Patulin Production by *P. expansum*

**FIGURE 1:**
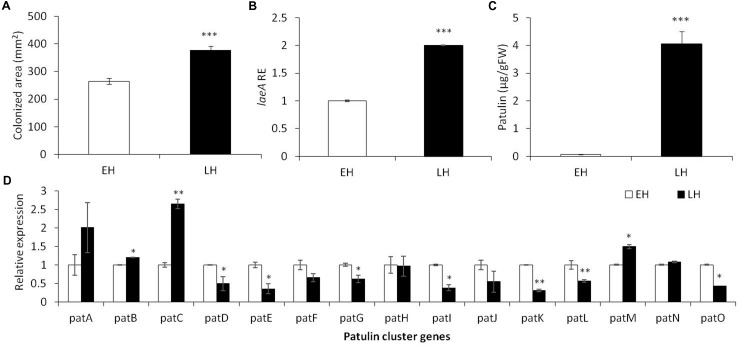
Effect of early (EH) and late harvested (LH) apple fruit on the relative expression of *laeA* and patulin cluster, patulin accumulation and colonized surface following the inoculation of *Penicillium expansum* in Golden Delicious fruits. Apple fruits cv. Golden Delicious were harvested first 135 days after fruit set (early harvest) and 156 days after fruit set (late harvest) and inoculated immediately. Five days later the colonization pattern **(A)**, *laeA* relative expression **(B)**, patulin accumulation in the colonized tissue **(C)**, and the relative expression of the *pat* cluster genes (PCG) **(D)** was evaluated. RNA was prepared from the leading edge of the colonized area. Average values (±SE) of five replicates are reported. For statistical analysis, the unpaired student *t*-test was used with significance defined as a *P* value (^∗^*P* ≤ 0.05; ^∗∗^*P* ≤ 0.01; ^∗∗∗^*P* ≤ 0.001).

To determine the effect of dynamic changes occurring during fruit maturation on patulin accumulation by *P. expansum* in Golden Delicious (GD) fruits, apples were inoculated at different periods during fruit maturation (**Table 1**). These results showed that a relatively higher amount of patulin was observed in more mature fruits. Comparison of “early” and “late” maturity harvested fruits showed a decrease of firmness by 3.42 lbs/cm^2^, an increase by 3.17% of total soluble solids (TSSs), a decrease by 0.12% in malic acid, and increase by 0.3% of pH values in more mature fruits. These changes were accompanied by an increase of the colonized area by 113 mm^2^ reflecting an increased susceptibility with maturity (**Figure 1A**).

**Table 1:** Physiological parameters of early and late harvested fruits.

Apple harvest	Firmness (lbs/cm^2^)	TSS (%)	Fruit pH	Acid content (%)
Early harvest	18.18 ± 1.35^∗∗^	13.75 ± 0.64^∗∗^	2.77 ± 0.05^∗∗^	0.46 ± 0.03^∗∗^
Late harvest	14.76 ± 1.37	16.92 ± 0.18	3.07 ± 0.04	0.38 ± 0.01

*^∗∗^Significant difference at the level of 0.05. Twenty freshly harvested fruits were included in the comparison.*

As *laeA* has recently been linked with *pat* gene cluster expression and patulin synthesis (Kumar et al., 2017), we also assessed *laeA* expression in the leading edge of infection of colonized fruits as well as the accumulation of patulin in the middle of the rotten tissue. A twofold increase in the relative expression of *laeA* and an increased accumulation of patulin from 0.07 to 4.05 μg/g/FWT were observed along with advanced maturation (**Figure 1B**).

Finally, we compared the relative expression of the 15 *pat* cluster genes involved in the biosynthesis of patulin in the two maturity apple maturity states (**Figure 1D**). We found a significant increase in the expression of *pat A, B, C*, and *M* in correlation with patulin synthesis in late harvested (more mature apples), but most of the cluster was down regulated in the LH fruits even when the amount of patulin was higher. These changes in expression were not large and we suggest it is possible that the production of patulin were the result of various post-transcriptional changes such as Pat protein activity/stability, patulin precursor availability, etc.

### Fruit Cultivar Affects Differential Expression of *pat* Genes and Consequent Patulin Production by *P. expansum*

**FIGURE 2:**
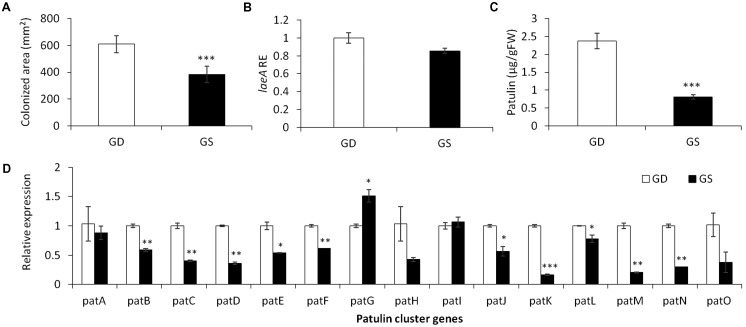
Effect of differential response of two apple cultivars, Golden Delicious (GD) and Granny Smith (GS) on the relative expression of *laeA* and patulin cluster, patulin accumulation, and colonized surface following the inoculation of *P. expansum*. Apple fruits were sampled from storage at 0°C and inoculated immediately. Five days later the colonization pattern **(A)**, *laeA* relative expression **(B)**, patulin accumulation in the colonized tissue **(C)**, and the relative expression of the *pat* cluster genes (PCG) **(D)** was evaluated. RNA was prepared from the leading edge of the colonized area. Average values (±SE) of five replicates are reported. For statistical analysis, the unpaired student *t*-test was used with significance defined as a *P* value (^∗^*P* ≤ 0.05; ^∗∗^*P* ≤ 0.01; ^∗∗∗^*P* ≤ 0.001).

To limit the amount of physiological parameters that may affect patulin accumulation during *P. expansum* colonization, we compared two different apple cultivars GD and Granny Smith (GS) at a similar level of firmness and TSS content but differing in the level of acidity and pH (**Table 2**). GD showing a higher pH value of 3.3 and lower acid content of 0.28% compared to GS cultivar with a pH of 2.9 and acid content of 0.57% had a bigger decay area from the fungus (610 compared 385 mm^2^) (**Figure 2A**) and showed a 1.3 fold increase in *laeA* expression, and threefold increase in patulin accumulation (2.4 compared 0.8 μg/g/FWT compared to the GS) (**Figures 2B,C**). The decrease in *laeA* expression and patulin accumulation (**Figures 2B,C**) in colonized tissue of GS by *P. expansum* occurred with the down-regulation of 10 *pat* genes (**Figure 2D**), a more consistent response that seen in maturity changes in GD (**Figure 1D**).

**Table 2:** Fruit physiological parameters of two different cultivars.

Apple cultivar	Firmness (lbs/cm^2^)	TSS (%)	Fruit pH	Acid content (%)
Golden Delicious	17.02 ± 1.2	13.5 ± 1.35	3.3 ± 0.09^∗∗^	0.28 ± 0.03^∗∗^
Granny Smith	17.59 ± 0.9	13.4 ± 1.37	2.90 ± 0.04	0.57 ± 0.01

*^∗∗^Significant difference at the level of 0.05. Twenty freshly harvested apples were included in the comparison.*

### Sucrose, Malic Acid and pH Differentially Regulate *laeA*, *pat* Genes and Patulin Production by *P. expansum*

**FIGURE 3:**
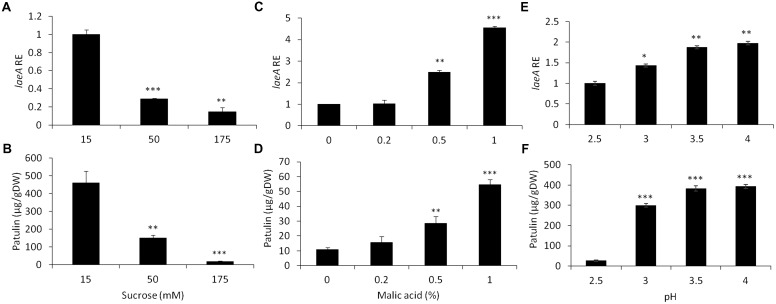
Effect of sucrose, malic acid, and pH changes on the relative expression of *laeA* and patulin accumulation. Solid SM media at initial pH 5.0 was amended different concentrations of sucrose and malic acid and the pH of the SM media also brought to different pH values. The solid SM media was inoculated with 100 μl of a 10^6^ spore/ml suspension and *laeA* relative expression **(A,C,E)** and patulin accumulation **(B,D,F)** and were analyzed were evaluated 3 days post-inoculation. Five 10-mm diameter disks were sampled from five independent culture plates. Average value (±SE) of five replicate are reported. Experiments were repeated three times and results of a single representative experiments are shown. For statistical analysis, the unpaired student *t*-test was used with significance defined as a *P* value (^∗^*P* ≤ 0.05; ^∗∗^*P* ≤ 0.01; ^∗∗∗^*P* ≤ 0.001).

Replicating previous studies (Kumar et al., 2017), we found both *laeA* expression and patulin were repressed in experiments where the fungus was grown in artificial media at increasing sucrose concentrations from 15 to 175 mM in standard growth medium (**Figures 3A,B**). The maximal expression of *laeA* and patulin accumulation were obtained when the fungus was grown in the presence of limiting sucrose level of 15 mM, while *laeA* RE decreased by sevenfold and the accumulation decreased by 95% (from 460 to 18.3 μg/gDW) in the presence of 175 mM sucrose (**Figure 3A**). These results are in contrast to the increase in both *laeA* expression and patulin accumulation in apples with increased maturity and sucrose accumulation. To try and understand why these results demonstrate seemingly opposite conclusions, we asked if other apple constituents might modify the impact of sucrose on both *laeA* expression and patulin production.

We first examined if the addition of malic acid, that is the main organic acid present in the apple flesh, affected *laeA* expression and/or patulin synthesis. **Figure 3** shows that increasing the concentrations of malic acid from 0 to 1% by itself, in the lack of any sucrose in the media, showed a 4.5 increase in RE of *laeA* and a fivefold increase of patulin accumulation (from 10 to 55 μg/gDW) (**Figures 3D,C**).

Another factor that might affect *laeA* expression and the accumulation of patulin is the pH change as it was found that pH in the fruit increased with apple maturation from 2.7 to 3 (**Table 1**). Thus, we next modified the pH of growth medium from 2.5 to 3.5 at a limiting sucrose level of 50 mM, and found a 1.9 fold increase of *laeA* RE and an increase of patulin accumulation by 10 fold with increasing pH (from 27 to 382 μg/gDW) (**Figures 3E,F**).

**FIGURE 4:**
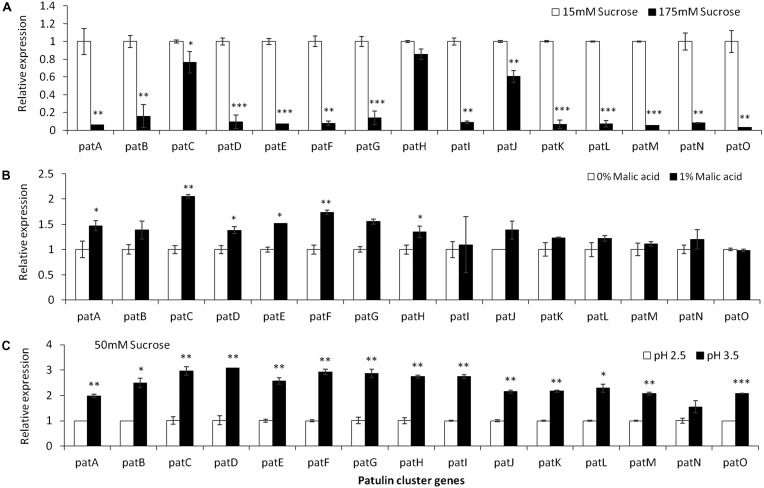
*pat* genes cluster (PGC) relative expression as affected by sucrose and malic concentrations and pH levels. Solid SM media at initial pH 5.0 amended with sucrose **(A)**, malic acid **(B)**, and various pH **(C)** on SM solid media were compared. The media was inoculated with 100 μl of a 10^6^ spore/ml suspension and the relative expression *patA, patB, patC, patD, patE, patF, patG, patH, patI, patJ, patK, patL, patM, patN, patO* between treatments were compared. Expression was evaluated 3 days post-inoculation after the mycelia was sampled from the culture plates. Average values (±SE) of five replicates are reported. Experiments were repeated three times and results of a single representative experiment are shown. For statistical analysis, the unpaired student *t*-test was used with significance defined as a *P* value (^∗^*P* ≤ 0.05; ^∗∗^*P* ≤ 0.01; ^∗∗∗^*P* ≤ 0.001).

Analysis of the relative expression of the *pat* genes in the presence of increased sucrose concentrations from 15 to 175 mM showed a strong decrease in expression (**Figure 4A**). This decrease was lost with the addition of 1% malic acid alone (**Figure 4B**). pH Changes in the presence of a limiting concentration of sucrose, 50 mM, also affected *pat* gene expression (**Figure 4C**).

### Combinatory Effect of Sucrose and Malic Acid on Patulin Accumulation by *P. expansum*

**FIGURE 5:**
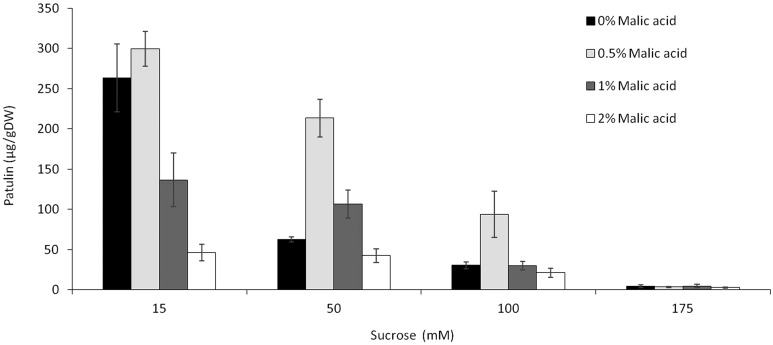
Effect of the combination of sucrose and malic acid in the SM media on patulin accumulation by *P. expansum*. Solid SM-media at initial pH 5.0 amended with concentrations of 15–175 mM sucrose in combination with 0–2% concentrations of malic acid. SM plates were inoculated with 100 μl of a 10^6^ spore/ml and patulin was evaluated 3 days post-inoculation. Five 10-mm-diameter disks were sampled from five independent culture plates. Average values (±SE) of five replicates are reported. Experiments were repeated three times and results of a single representative experiment are shown.

Given that sugars and organic acids are available in fruits at dynamic quantities during maturation and ripening, we tested the combination of different sucrose and malic acid at a fixed pH 5. Overall, the accumulation of patulin by *P. expansum* as a function of malic acid content appears to have a bell-shaped distribution with an optimum accumulation at 0.5% malic acid in the medium (similar to the concentration found in fresh apples) at all three tested sucrose levels of 15, 50, and 175 mM (**Figure 5**). A further increase of malic acid content to 1 and 2% resulted in a decrease of patulin accumulation level at all three tested sucrose concentrations. Interestingly, the increase of sucrose level to 175 mM resulted in a complete inhibition of patulin accumulation independently of the malic acid content (**Figure 5**), indicating that sucrose is the leading factor modulating patulin accumulation in growth media as malic acid showed no effect at higher sucrose concentrations.

### Fruit Phenols Differentially Regulate *laeA*, *pat* and Patulin Production by *P. expansum*

#### Chlorogenic Acid

**FIGURE 6:**
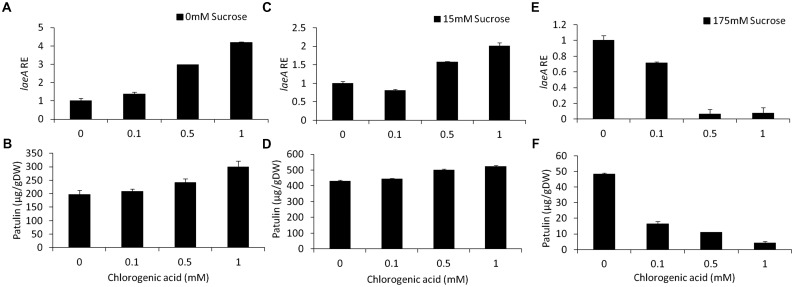
Effect of chlorogenic acid in absence and presence of different sucrose levels on relative gene expression and patulin accumulation. Solid SM media at initial pH 5.0 were amended with 0, 15, 175 mM sucrose and combined with chlorogenic acid at concentrations ranging from 0 up to 1 mM. The effect of the combination of sucrose/chlorogenic was tested on *laeA* relative expression (RE) **(A,C,E)** and patulin accumulation **(B,D,F)**. The media was inoculated with 100 μl of a 10^6^ spore/ml suspension. RE of *laeA* and patulin accumulation were evaluated on the third day post-inoculation. Five 10-mm diameter disks were sampled from five independent culture plates. Average values (±SE) of five replicates are reported. Experiments were repeated three times and results of a single representative experiment are shown.

**FIGURE 7:**
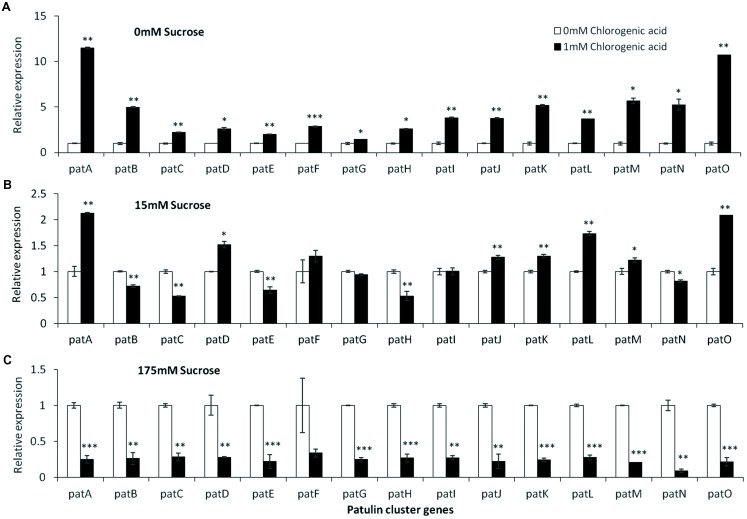
*pat* genes cluster (PGC) relative expression as affected by chlorogenic acid and different levels of sucrose. Solid SM media at initial pH 5.0 amended with 0 **(A)**, 15 **(B)**, and 175 mM **(C)** sucrose was combined with increasing concentrations of chlorogenic acid from 0 and up to 1 mM. The media was inoculated with 100 μl of a 10^6^ spore/ml suspension and the relative expression *patA, patB, patC, patD, patE, patF, patG, patH, patI, patJ, patK, patL, patM, patN, patO* between both treatments were compared. Expression was evaluated 3 days post-inoculation after the mycelia was sampled from the culture plates. Average values (±SE) of five replicates are reported. Experiments were repeated three times and results of a single representative experiment are shown. For statistical analysis, the unpaired student *t*-test was used with significance defined as a *P* value (^∗^*P* ≤ 0.05; ^∗∗^*P* ≤ 0.01; ^∗∗∗^*P* ≤ 0.001).

Chlorogenic acid is a phenolic compound present in apples at an average concentration of 170 mg/kg FW (Burda et al., 1990; van der Sluis et al., 2001). In our *in vitro* experiments, increasing chlorogenic acid concentrations in solid media from 0 to 1 mM in the absence of sucrose in the medium, showed a fourfold upregulation of *laeA* together with an increase of patulin concentrations from 197 to 299 μg/gDW (**Figures 6A,B**). On the other hand, increasing the concentrations of chlorogenic acid in the presence of limited (15 mM) and excess (175 mM) sucrose levels, showed different responses. In the presence of 15 mM sucrose and the absence of chlorogenic acid, the level of patulin accumulation was 430 μg/gDW (**Figure 6D**). When the concentration of chlorogenic acid in the medium was adjusted to 1 mM, patulin production increased *laeA* RE showed a twofold increase and 18% increase in patulin accumulation was observed (**Figures 6C,D**). At 175 mM sucrose the addition of chlorogenic acid further decreased *laeA* expression and patulin accumulation (**Figures 6E,F**). *pat* Gene expression followed suit, where chlorogenic acid increased gene expression with no sucrose, had little impact with 15 mM sucrose and decreased *PAT* gene expression in 175 mM sucrose (**Figure 7**).

These results indicate that sucrose overrides the chlorogenic acid effect in these conditions and that the relative expression of *laeA* and *pat* genes and concomitant patulin accumulation may be differently modulated by single and complex mixtures of nutritional components like chlorogenic acid and sugar present in fruits.

#### Epicatechin

**FIGURE 8:**
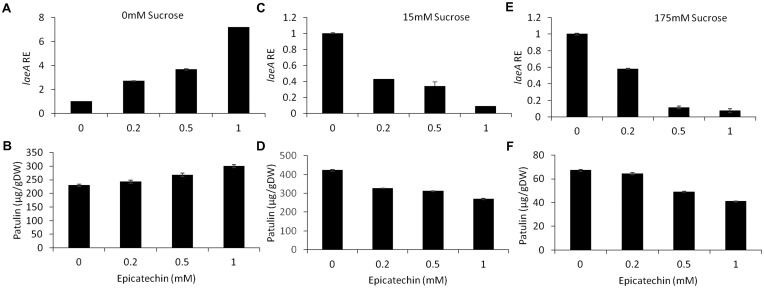
Effect of epicatechin in absence and presence of different sucrose levels on patulin accumulation and *laeA* relative expression. Solid SM media at initial pH 5.0 were amended with 0, 15, 175 mM sucrose and combined with epicatechin at concentrations ranging from 0 up to 1 mM. The effect of the combination of sucrose/epicatechin was tested on *laeA* relative expression (RE) **(A,C,E)** and patulin accumulation **(B,D,F)**. The media was inoculated with 100 μl of a 10^6^ spore/ml suspension. Patulin and *laeA* relative were evaluated on the third day post-inoculation. Five 10-mm diameter disks were sampled from five independent culture plates. Average values (±SE) of five replicates are reported. Experiments were repeated three times and results of a single representative experiment are shown.

**FIGURE 9:**
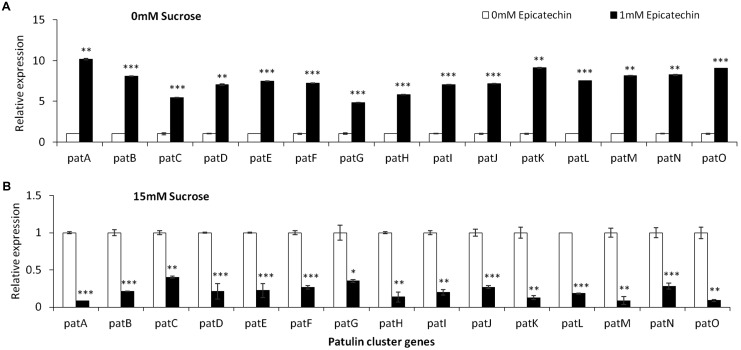
*pat* genes cluster (PGC) relative expression as affected by epicatechin and different levels of sucrose. Solid SM media at initial pH 5.0 amended with 0 **(A)**, and 15 **(B)** sucrose was combined with increasing concentrations of chlorogenic acid from 0 and up to 1 mM. The media was inoculated with 100 μl of a 10^6^ spore/ml suspension and the relative expression *patA, patB, patC, patD, patE, patF, patG, patH, patI, patJ, patK, patL, patM, patN, patO* between both treatments were compared. Expression was evaluated 3 days post-inoculation after the mycelia was sampled from the culture plates. Average values (±SE) of five replicates are reported. Experiments were repeated three times and results of a single representative experiment are shown.

Epicatechin is a flavonoid, another subgroup of phenolic compounds found in woody plants and apples, with amounts ranging between 66 and 71 mg/kg FW in the flesh of GD apples (Lee et al., 2003; Tsao et al., 2003). Amendment with epicatechin gave an overall similar result as chlorogenic acid: there was a trend toward an increase in, *laeA* expression, *pat* cluster expression and patulin when no sucrose was present but when sucrose was present (both 15 and 175 mM), gene expression and patulin synthesis decreased (**Figures 8**, **9**).

Together, these results suggest that natural phenols present in the fruit may modulate patulin biosynthesis in *P. expansum* at the transcriptional level.

## Discussion

Apple fruits are colonized by the mycotoxigenic *P. expansum* through entry via damaged surfaces—mechanical injuries, insect wounds, cuts, and splits—that occur during harvesting, storage packing, transporting, and marketing. Fruit colonization is accompanied by the synthesis of the mycotoxin patulin. Some studies suggested that patulin accumulation contributes to pathogenicity of *P. expansum* (Barad et al., 2015), some indicated that the patulin accumulation has no effect at all on virulence (Ballester et al., 2015) and other research reported that patulin produced by *P. expansum* is a cultivar-dependent aggressiveness factor favoring the colonization of apples (Snini et al., 2016). This differential cultivar response led us to analyze in two dissimilar varieties, the possible host internal factors that may modulate patulin accumulation.

### Fruit Maturity Enhances *laeA* Expression and Patulin Accumulation

Comparison of fruits at different stages of maturity of the susceptible cultivar GD showed that as the fruit become more mature with increasing TSS values, reduced malic acid content and reduced firmness, it showed an enhanced susceptibility to *P. expansum* infection. This increased susceptibility was accompanied by a small yet significant increase in expression of *laeA* and in patulin accumulation, however, with little correlation with *pat* gene expression. A more consistent pattern of *pat* gene expression was observed in the GS cultivar. In this case, the reduced patulin accumulation observed in GS was accompanied with almost all *pat* genes being downregulated (**Figure 2**). Likely both transcriptional and post-transcriptional and/or patulin precursor availability explain some of these differences.

### Sucrose, Malic Acid and pH Differentially Modulate *laeA* Expression and Patulin Accumulation

Apple fruits contain many nutrients including sucrose and malic acid. While *laeA* and patulin accumulation were negatively affected by the increase of sucrose content from 15 to 175 mM, addition of malic acid and/or modulation of pH affected these outcomes. Previous work had shown the impact of sucrose and pH on *laeA* and *pat* gene expression as well as patulin synthesis but only as sole parameters (Kumar et al., 2017). A similar response was observed when the most active inducer of patulin production, malic acid, and the most active inhibitor, sucrose, were combined in media (**Figure 5**). Here, again the higher sucrose concentrations override the effect of malic acid under *in vitro* conditions.

However, the increase of sucrose level during fruit ripening didn’t show the same effect observed *in vitro* (**Figure 1**), suggesting that other factors are interfering and overriding the effect of sucrose alone. In this regard, Vilanova et al. (2012) have showed that fruit ripening is a complex process involving a variety of biochemical changes that is associated with increased susceptibility to *P. expansum*. In that study, lignin accumulation at the infection site was higher in apples harvested prior to maturity and suggested to be an essential component of resistance against *P. expansum* (Vilanova et al., 2012). Moreover, other plant defense mechanisms were found to be fruit maturity-dependent. The generation and accumulation of H_2_O_2_ was significantly higher in early harvested apples, contributing to the resistance of these fruits to fungal infection. We believe that these changes in the host resistance observed during fruit ripening mask the effect of the natural apple components and contribute to fungal attack and potentially to patulin accumulation.

The effect of malic acid was completely different, showing an enhanced activation of *laeA* and patulin accumulation *in vitro*. This is putatively resulting from the acidification process induced by malic acid that contribute to an increased expression of *laeA* and *pat* genes and consequently to a higher patulin accumulation. This response should normally be enhanced under *in vivo* conditions due to the accumulation of another organic acid, gluconic acid that contributes additionally to the acidification process and to the accumulation of patulin (Barad et al., 2014). However, this was not observed in our *in vivo* experiments, where the GS apple cultivar containing higher amounts of malic acid (Nour et al., 2010) with similar TSS % compared to GD cultivar—showed reduced colonization and patulin accumulation (**Figure 2**). This reflects again the complexity of interactions between the different apple intrinsic factors resulting in different effects than those observed *in vitro*. The *in vivo* results presented here are in disagreement with those reported previously by Snini et al. (2016). In the latter study, the GS cultivar was found to be more sensitive to *P. expansum* infection compared to the GD cultivar, as the production of patulin was found not essential to promote progression of the fungus within the GS apple tissue. This suggests that a future analysis of the intrinsic fruit factors affecting patulin of a wider amount of cultivars should be taken in account.

### Chlorogenic Acid and Epicatechin in Combination With Sucrose Differentially Modulate *laeA* Expression and Patulin Synthesis

Chlorogenic acid and epicatechin are also nutrition factors in apples. However, their observed effect on the activation of *laeA* and the patulin cluster indicated that they may become signaling compounds for important processes leading to patulin accumulation. Chlorogenic acid and epicatechin were showed to induce *laeA* expression accompanied with 4–10 fold increase of *pat* expression, respectively (**Figures 6**, **8**). However, this induction of *laeA* expression by epicatechin and chlorogenic acid was strongly inhibited by the presence of excess sucrose in the medium. This sugar was found to overrides the effect of both phenols leading to a full inhibition of *pat* expression and consequently the inhibition of patulin accumulation. The findings reported here on the enhancement of patulin production by both tested phenolic compounds were a little surprising as many previous reports showed a negative impact of phenolic compounds on fungal growth and mycotoxin accumulation (Samapundo et al., 2007; Telles et al., 2017). The mechanism by which these phenols modulate the whole process is still unknown but indicate the potential importance of these molecules in the regulation of fungal metabolism during *P. expansum* colonization.

## Conclusion

The findings in this study provide an initial insight into fruit intrinsic factors that may have a significant impact on *P. expansum’s* capability to produce and accumulate patulin in apple, however, this should be widened to more cultivars to confirm this new findings. The correlation of increased sucrose and patulin production in apples was not observed in growth media suggesting that other metabolites in the host have a large impact on patulin synthesis by the fungus. We show here that combinations of apple nutrients can alter the impact of sucrose on *laeA* and *pat* expression as well as patulin production *in vitro* and suggest a similar but even more complex affect occurs in fruit.

## Materials and Methods

### Fungal Strain, Culture Conditions, and Host

Wild type strain of *P. expansum*, isolate Pe-21, was obtained from decayed apples (*Malus domestica* cv. GD) as described by Hadas et al. (2007). Cultures were grown at room temperature in the dark, and maintained on PDA plates (Difco) unless otherwise indicated. Conidia were harvested with 10 ml of sterile distilled water supplemented with 0.01% (v/v) Tween 80 (Sigma-Aldrich). Cells were visualized with a model BX60F-3 microscope (Olympus America, Inc.) and counted using a hemocytometer.

### Host Physiological Parameters and Inoculation Conditions

Golden Delicious’ apples were freshly harvested from three trees in a single orchard in North Israel (Rosh Pina) starting 09 September, 2016, approximately 135 days after fruit set (early harvest). Second harvest or “late harvest” samples were collected at intervals up to 21 days on 22 September, 2016. Harvested fruits were analyzed for firmness, acidity, pH, and TSS as described below. On the day of harvest, fruits were wounded and inoculated with 5 μl of the wild type strain Pe-21 spore suspension (1 × 10^6^ spores/ml). On a 2 mm depth and incubated under high humidity at room temperature. GD and GS apples, having similar firmness level and TSS content but different acid levels (%), were chosen from the storage house “Bereshit.”

Fruit firmness was determined by punching both sides of each fruit using a penetrometer (Lutron FG-20KG, Made in Taiwan) fitted with an 11 mm diameter flat plunger. Measurements were carried out at two equatorial opposite positions on each side of 10 apples and results were expressed in lbs/cm^2^. To determine TSS content in fruits, juice from 20 apples (100 g tissue from each apple in four replicates) was extracted using an electric juicer (Maulinex juice extractor, Model M833) and filtered using Gauze pad. TSS content (%) in juice was measured by a digital refractometer (Atago, Tokyo, Japan). Subsequently, acid content and pH of fruit juice were determined using Metrohm titrator (678 EP/KF processor), Switzerland.

### Patulin Accumulation

To evaluate the patulin accumulation capability of the Pe-21 strain of *P. expansum* under different culture conditions, 100 μl of a 10^6^ spore/ml spore suspension) were used to inoculate 55-mm diameter petri dishes containing 10 ml of solid SM media containing (per liter): 7 g NaNO_3_, 3 g tryptone (Difco), 1 g KH_2_PO_4_, 0.5 g MgSO_4_⋅7H_2_O, and 0.5 g KCl, different concentrations of sucrose (15, 50, 100, and 175 mM, as indicated in each experiment), different concentrations of malic acid (0, 0.5, 1, and 2%), different pH (2.5, 3, 3.5, and 4) levels at 50 mM sucrose, different concentrations of chlorogenic acid (0, 0.1, 0.5, and 1 mM), various concentrations of epicatechin (0, 0.2, 0.5, and 1 mM) and 2% agar adjusted to pH 5, with concentrated HCl. In experiments that don’t indicated the amount of malic acid, chlorogenic acid, and epicatechin are considered as 0. The plates were incubated at 25°C in the dark for 72 h. Mycelium was peeled off the plates, frozen in liquid nitrogen and lyophilized for RNA extraction. Five 1 cm^2^ diameter disks of SM-agar were placed in 5 ml sterilized water and homogenized with an HG-300 homogenizer (MRC). To analyze the parameters as dry weight (DW), three whole medium plates were heated in a microwave, the agar was soaked up with a paper towel and the remaining mycelia were lyophilized for 24 h. The DW was measured on an analytical scale (Sartorius, Göttingen, Germany).

To analyze patulin accumulation in apple colonized tissue, the same fresh weight of each decay area was taken, 5 ml of DDW was added and the tissues were homogenized. The final pH in the homogenized samples from *in vivo* and *in vitro* experiments was measured using a double-pore slim electrode (Hamilton) connected to a Thermo Orion Model 720A Plus pH meter. Patulin accumulation in the homogenized agar disk plates or apple tissue was evaluated as described by Barad et al. (2014). Briefly, patulin was extracted by adding 10 ml of ethyl acetate to the homogenized samples than vortexing for 1 min. Samples were later centrifuged for 5 min at room temperature at 4,000 ×*g* and the upper organic phase was transferred to fresh tube and washed with 10 ml of a 1.5% sodium carbonate solution After centrifugation for 5 min at 4,000 ×*g*, organic phases were collected and left to dry under a fume hood. Completely dried samples were re-dissolved in 0.5 ml of the elution solution containing 0.02 M ammonium acetate and acetonitrile (9:1, vol/vol), and filtered through a 0.22 μm Minisart filter (Sartorius Stedim, Göttingen, Germany). Quantitative analysis of patulin was performed by a high-performance liquid chromatograph (HPLC) (Hitachi-Merck, Dartford, United Kingdom) equipped with a UV-VIS detector at 280 nm. Fifty microliters of each sample were injected to the HPLC equipped with a C18, 250-by-4.6-mm, Microsorb-MV-100-5 column, at a rate of 0.8 ml/m. The patulin fraction was eluted with 0.02 M ammonium acetate and acetonitrile (9:1, vol/vol) with a retention time of approximate 7 min. Results were compared with a commercial patulin standard (Sigma-Aldrich).

### Nucleic Acid Analysis

RNA extraction was carried out using SV Total RNA Isolation kit (Promega). Purity of the extracted RNA was evaluated using aND-1000 spectrophotometer (NanoDrop Technologies, Inc.), and samples were stored at −80°C until further analysis. Total RNA was extracted from apple fruits according to Yang et al. (2008), with minor changes; aliquots were taken from pooled samples from the leading edges of the four inoculation areas of each apple as described earlier by Barad et al. (2016).

### Gene-Expression Analysis by qRT-PCR

To analyze gene expression, RNA was extracted and the reverse-transcription reaction was performed on 1 μg of total RNA with the Reverse-it First-Strand Synthesis Kit (ABgene) according to the manufacturer protocol, and cDNA samples were diluted 1:10 (v/v) with ultrapure water.

Real-time qPCR was performed with the StepOnePlus System (AB, Applied Biosystems, Singapore). PCR amplification was performed with 3.4 μl of cDNA template in a 10 μl reaction mixture containing 6.6 μl of SYBR Green Amplification kit (ABgene) and 300 nM primers. PCR was carried out with the following cycling program: 10 min at 94°C, followed by 40 cycles of 94°C for 10 s, 60°C for 15 s, and 72°C for 20 s. The samples were subjected to melting-curve analysis, with efficiencies close to 100% for all primer pairs, and all products showed the expected size of 70–100 bp. All samples were normalized to 28S expression levels and the values were expressed as the change in increase or decrease of the relative levels of a calibrator sample. Results were analyzed with StepOnePlus v.2.2.2 software. Relative quantification was performed by the ΔΔC_T_ method (Livak and Schmittgen, 2001). The ΔC_T_ value was determined by subtracting the C_T_ results for the target gene from those for the endogenous control gene and normalized against the calibration sample to generate the ΔΔC_T_ values. Each experiment was performed in triplicate, and three different biological experiments were conducted. One representative set of results are presented as mean values of 2^−ΔΔC^_T_ ± SE for each treatment. All primers and their sequences are listed in (Supplementary Table 1).

### Statistical Analysis

Data were analyzed with the JMP software package, version Pro10 (SAS Institute). Mean comparisons of gene expression, patulin production and ambient pH measurements were analyzed according to least significant difference. For statistical analysis, the unpaired student *t*-test was used with significance defined as a *P*-value (^∗^*P* ≤ 0.05; ^∗∗^*P* ≤ 0.01; ^∗∗∗^*P* ≤ 0.001).

## Author Contributions

DP evaluate the concept and wrote the manuscript. NK reviewed the manuscript and suggested experiments. JT reviewed the manuscript and suggested experiments. ES reviewed the manuscript and contributed project submission and DK did significant part of the work.

## Conflict of Interest Statement

The authors declare that the research was conducted in the absence of any commercial or financial relationships that could be construed as a potential conflict of interest.

## Supplementary Material

TABLE S1 Nucleotides used for qPCR study.
