# Supplementary material for: Apple Intrinsic Factors Modulating the Global Regulator, LaeA, the Patulin Gene Cluster and Patulin Accumulation During Fruit Colonization by Penicillium expansum
Source: Front Plant Sci. 2018 Jul 27;9:1094. doi: 10.3389/fpls.2018.01094 (PMC6073165; doi:10.3389/fpls.2018.01094)
Supplement: TABLE S1 — Nucleotides used for qPCR study. [file Table_1.docx]

**Supplementary table 1**. Nucleotides used for qPCR study

|  | **Oligoucleotides** | **Nucleotide sequences (5′-3′)** |
| --- | --- | --- |
|  | 28SF  28SR  LaeAF  LaeAR  patA_F-RT  patA_R-RT  patB_F-RT  patB_R-RT  patC_F-RT  patC_R-RT  patD_F-RT  patD_R-RT  patE_F-RT  patE_R-RT  patF_F-RT  patF_R-RT  patG_F-RT  patG_R-RT  patH_F-RT  patH_R-RT  patI_F-RT  patI_R-RT  patJ_F-RT  patJ_R-RT  patK_F-RT  patK_R-RT  patL_F-RT  patL_R-RT  patM_F-RT  patM_R-RT  patN_F-RT  patN_F-RT  patO_F-RT  patO_R-RT | GGAACGGGACGTCATAGAGG  AGAGCTGCATTCCCAAACAAC  TGGTAGTGTCGCAAGTTGGC  TCGACCTGCTCAAACCAGG  AAAGGCCGGTGCATTGATC  TTGGAGGCTTTGGTGAGCAT  GCCAGGCTATGCGATTGAGT  GCTGGAACCCTGTCCATTGT  TCCACCTGCGAATATCCCTTA  CATCGCCAGTGCCATTTTC  ATGAGATTCGTCTGCGCAAAG  CTACCCAAGCGGGATGAGATT  CATTCTCATCGGGCCTGAGT  TCGAAGCTCTTCCGGACATG  GCGAGTGAATTCGGCCAAT  GTCCGACCCAAAGGATGAAG  CGGCCGTCTTGAAGGAAAT  CTTGCCGTAGCGGGTGAATA  CATTTATCGGCGGTGTTCTGA  GATCAACGCTTGCACGATAGC  GCAAACTCATTCCGCAAGGA  TGGTTCTTGCCATCGATCAC  CGCCAGACATACCGCCATA  TTTGGTCGATCGGGACTGTT  GACGCTGGGCTACTGGATTG  TCGTGCGTGAGGCCAGTAT  GCAGGAGATCCGTTTCAGACA  CCACTGACCGACGGTTACAAC  ACCCACAGCTGCACATGGA  AGCGAGAAGAGGCGGAAGA  CGTTCGATGTCGCTAGCAAA  GGCGATAATCACGTCAATTCG  TCGCCTCCTGGTGTGTATCTT  AAGCGTGCCCAGTCATTCAG |
